# Stability of *Burkholderia cepacia* Lipase Immobilized on Styrene–Divinylbenzene Activated with Glutaraldehyde, Triton X-100, and Polyethylene Glycol for the Green Synthesis of Hexyl Acetate

**DOI:** 10.1007/s12010-025-05277-2

**Published:** 2025-06-04

**Authors:** Wellington M. Correa, Wislei R. Osório, Ausdinir D. Bortolozo, Erik Poloni, Giovana S. Padilha

**Affiliations:** 1 Centro de Pesquisa em Manufatura e Materiais Avançados, Faculdade de Ciências Aplicadas, Universidade Estadual de Campinas (Unicamp), Limeira, SP 13484-350 Brazil; 2 Faculdade de Tecnologia, Universidade Estadual de Campinas (Unicamp), Limeira, SP 13484-332 Brazil; 3 Department of Materials, Imperial College London, London, SW7 2 AZ UK; 4 Present Address: School of Engineering and Materials Science, Queen Mary University of London (QMUL), London, E1 4 NS UK

**Keywords:** Physical adsorption, Chemical additives, Pear flavor synthesis

## Abstract

This work focuses on improving the stability of *Burkholderia cepacia* lipase immobilized on styrene–divinylbenzene by using chemical additives and a cost-effective physical adsorption method. Ethanol pretreatment of the supports proved essential for maintaining enzyme activity. The optimal conditions for immobilization were achieved at a 1:1 support-to-enzyme ratio, pH 8, 200 rpm, and 60 °C. Combinations of the additives glutaraldehyde, polyethylene glycol 1500, and Triton X-100 were examined for activation treatment of supports before immobilization. Concentrations of 2.5% (w/v) of polyethylene glycol 1500 and 0.5% (v/v) of Triton X-100 were used to maximize biocatalyst activity. We show that the activated biocatalyst yielded up to 950% more hexyl acetate than non-activated control after 12 reaction cycles. Fourier transform infrared spectroscopy and scanning electron microscopy confirmed the effective immobilization of the *Burkholderia cepacia* lipase. This study introduces a scalable and sustainable method for creating robust biocatalysts aimed at producing value-added chemicals, thereby advancing green chemistry in the flavor industry.

## Introduction

Lipases (E.C. 3.1.1.3) are enzymes known for their ability to catalyze the hydrolysis of ester bonds. They have been extensively studied since the early twentieth century, and recent research has explored their applications in industries such as detergent production, fat and oil modification, flavor and food processing, pharmaceuticals, and biofuels [1–8]. The ability of lipases to operate in moderate environments reduces energy consumption and waste generation, indicating a more environmentally friendly substitute to conventional chemical catalysts [9–11]. Notably, traditional chemical synthesis typically requires aggressive catalysts, organic solvents, and energy-intensive processing routes, leading to lower selectivity, hazardous byproducts, and environmental and safety concerns in the food and fragrance industries [12]. The growing emphasis on sustainable practices in various industries has led to an increased utilization of biocatalysts, supporting the concept of a strong bioeconomy [13]. The global enzyme market is projected to experience significant expansion due to a rising demand for green chemistry solutions.

Although they are widely used, free lipases face challenges in industrial applications, including limited stability and reusability, and difficulty in separating from reaction mixtures. The immobilization of lipases on solid supports addresses these issues by enhancing stability, facilitating separation, and promoting reusability. This approach improves the economic and environmental viability of lipases [1, 4, 14–16]. Various methods exist for lipase immobilization, including enzyme entrapment [15, 16], physical adsorption [2], covalent binding [7], and others [6, 10, 17, 18]. Physical adsorption is a simple and economical method, but it can lead to enzyme leaching from the support surface during bioproduct synthesis. An effective approach to enhance the performance of lipases immobilized through physical adsorption is the use of chemicals such as glutaraldehyde (GLU), polyethylene glycol 1500 (PEG), and Triton X-100 (TX) for activation before immobilization [7, 9, 10, 18–22]. The addition of GLU is commonly known to promote the formation of covalent bonds between the enzyme and the support, enhancing immobilization stability and providing a protective framework around the enzyme [9, 18, 19, 21, 23]. PEG is considered an enzyme stabilizer, as it helps avoid enzyme segregation and promotes its diffusion. This stabilizing effect maintains structural integrity, contributing to improved performance and durability in catalytic applications [10, 20, 24, 25]. The use of TX stabilizes the enzyme structure and protects it from denaturation, while also improving its solubility and dissociating bimolecular aggregates in the immobilization solution, which leads to a more uniform distribution on the support material [25, 26].

Researchers have employed both natural and synthetic substances as supports for enzyme immobilization. Correa et al. [2], Dizge, Keskinler, Tanriseven [27], Aybastıer and Demir [28], and Bento et al. [29] have specifically highlighted the use of compounds derived from styrene–divinylbenzene copolymers (ST–DVB) to immobilize lipases. ST–DVB has high surface area and mechanical strength, making it an excellent choice for immobilization through physical adsorption. Because of its hydrophobicity, it tends to bind to hydrophobic lipases. Hydrophobic supports also enhance the stability of lipases when they are in an open-lid configuration [28, 30]. Although GLU, PEG, and TX are known to enhance enzyme immobilization efficiency through physical adsorption, their combined use has not yet been studied for immobilizing *Burkholderia cepacia* lipase (LBc) onto ST–DVB and optimizing the synthesis of bioproducts.

**Fig. 1:**
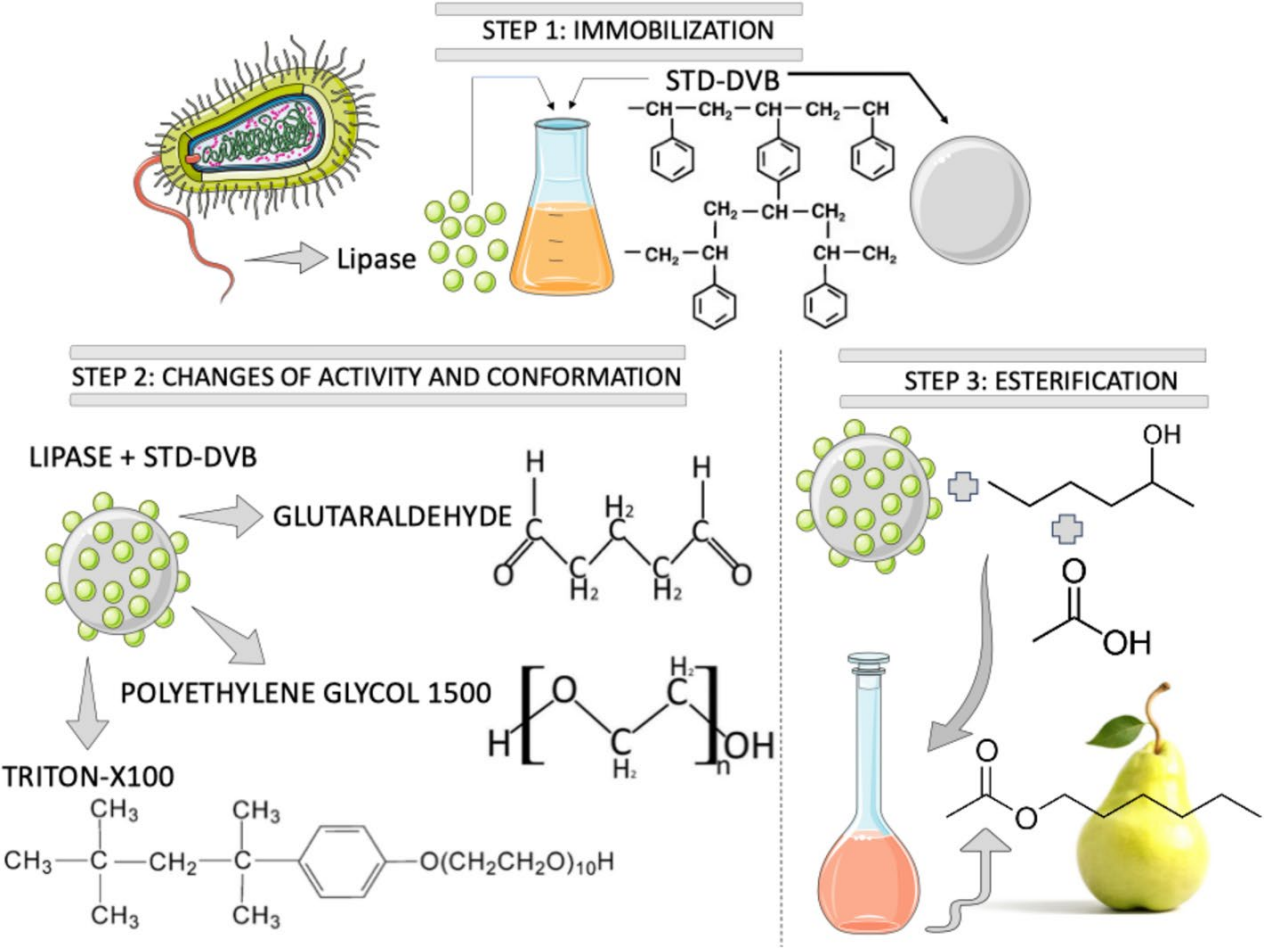
Schematic of this work, involving lipase immobilization, support activation, and esterification

In this study, we investigate the immobilization of LBc onto ST–DVB supports through physical adsorption. We explore the optimization of the immobilization parameters, and the influence of PEG, TX, and GLU combinations on support activation. Our results demonstrate that the optimized biocatalyst effectively produces hexyl acetate, a key compound in pear flavor. An overview of this work is shown in Fig. 1. This research advances the development of sustainable and efficient biocatalytic technologies, supporting environmentally friendly innovations in the flavor industry.

## Materials and Methods

### Materials

Commercial lipase from *Burkholderia cepacia* (LBc), styrene–divinylbenzene copolymer (ST–DVB, Diaion HP-20), acetic acid, Coomassie brilliant blue, 1-hexanol, hexane, heptane, molecular sieves beads Type 3 A, and pure hexyl acetate (analytical standard for calibration method via chromatography analysis) were purchased from Sigma-Aldrich, USA. Acetone, anhydrous citric acid, absolute ethanol (99.5%, v/v), sodium bicarbonate, potassium hydrogen phthalate, sodium carbonate, sodium citrate, dibasic sodium phosphate, potassium hydroxide, polyethylene glycol 1500 (PEG), and Triton X-100 (TX) were bought from Synth, Brazil. Gum arabic was obtained from Oxoid, UK, and monobasic sodium phosphate and alcoholic solution of phenolphthalein (1%, w/v) from Dinâmica, Brazil. Glutaraldehyde (GLU) was purchased from Vetec, Brazil. All chemicals were acquired at analytical grade and used without further purification. Olive oil (Carbonell) with maximum acidity of 0.5% was bought from the local market. Deionized water was used in all experimental stages.

### Lipase Immobilization Procedures

The styrene–divinylbenzene copolymer (ST–DVB) support underwent initial pre-treatment with ethanol to eliminate residual monomer impurities [31]. To this end, 10 g of ST–DVB particles was immersed in 95% (v/v) ethanol and agitated at 200 rpm at 27 ± 2 °C, in the absence of light, for 18 h. Next, the support was thoroughly washed with deionized water to remove all traces of ethanol, followed by vacuum filtration using a Büchner funnel. The particles were then dried under a stream of air at room temperature (27 ± 2 °C) for 12 h and stored for later use. Two different procedures were used for the lipase immobilization:

1. ST–DVB + lipase (immobilization without activation): following ethanol pre-treatment, 10 g of ST–DVB were added to 20 mL of *Burkholderia cepacia* lipase (LBc) solution with protein loading (30 mg.g^−1^), which had been previously diluted in 5 mM sodium phosphate buffer (pH 7). The mixture was homogenized at 250 rpm for 10 min at 25 ± 2 °C. The lipase immobilization was carried out using different parameters for optimization (see “Optimization of Lipase Immobilization Parameters” section). The biocatalysts were recovered by vacuum filtration, washed with deionized water to remove unbound lipase, and stored at 5 ± 1 °C for 24 h prior to use.
2. ST–DVB + lipase (immobilization with activation): After ethanol pre-treatment, the ST–DVB beads were submerged in solutions containing: (i) 5% of PEG, (ii) 1% of TX, (iii) 2.5% of GLU, (iv) 2.5% of PEG and 0.5% of TX, (v) 5% of PEG and 1% of TX, (vi) 2.5% of PEG and 1.25% of GLU, (vii) 5% of PEG and 2.5% of GLU, (viii) 0.5% of TX and 1.25% of GLU, (ix) 1% of TX and 2.5% of GLU, (x) 2.5% of PEG, 0.5% of TX and 1.25% of GLU, (xi) 5% of PEG, 1% of TX and 2.5% of GLU. The concentrations of PEG, TX and GLU are indicated in w/v, v/v and v/v, respectively. For chemical activation, the support suspensions were stirred at 200 rpm for 1 h at 27 ± 2 °C. After activation, the supports were recovered through filtration and thoroughly washed with cold hexane to remove all traces of unbound additives. Next, the supports were dried at 27 ± 2 °C for 12 h under circulating air. The immobilization was conducted using optimized parameters (see “Optimization of Lipase Immobilization Parameters” section). The biocatalysts were recovered by vacuum filtration, washed with deionized water, and stored at 5 ± 1 °C for 24 h prior to use.

### Optimization of Lipase Immobilization Parameters

Different values of lipase loading per unit of ST–DVB, agitation profile, pH, and temperature were used to optimize the immobilization of lipase. To study the lipase loading per unit of ST–DVB, ratios of 1:0.5, 1:1, 1:2, and 1:4 w/w were used along with an agitation of 150 rpm, temperature of 50 ± 2° C, and pH 7. A temperature variation within the range of 20 to 70 °C was investigated with a 1:1 w/w ratio of lipase loading to ST–DVB, an agitation of 200 rpm, and pH 7. The Vmax values were obtained at different temperatures to determine activation energies using the Arrhenius plot. The ideal gas constant value (8.31451 J.mol^−1^ K^−1^) was used to determine the activation energy for olive oil hydrolysis. The agitation profile was varied from 50 to 250 rpm while the lipase loading per unit of ST–DVB, temperature, and pH were kept, respectively, at 1:1 w/w, 50 ± 2 °C, and pH 7. The impact of pH was studied using buffers ranging from pH 3 to 10 (100 mM), including citrate buffer (pH 3 to 5), sodium phosphate buffer (pH 6 to 8), and carbonate buffer (pH 9 and 10), at a 1:1 lipase to support ratio, 200 rpm, and 60 ± 2 °C. pH stability was assessed by measuring residual activity at pH 6, 8, and 10, with samples stored at 5 ± 2 °C for 45 days.

**Fig. 2:**
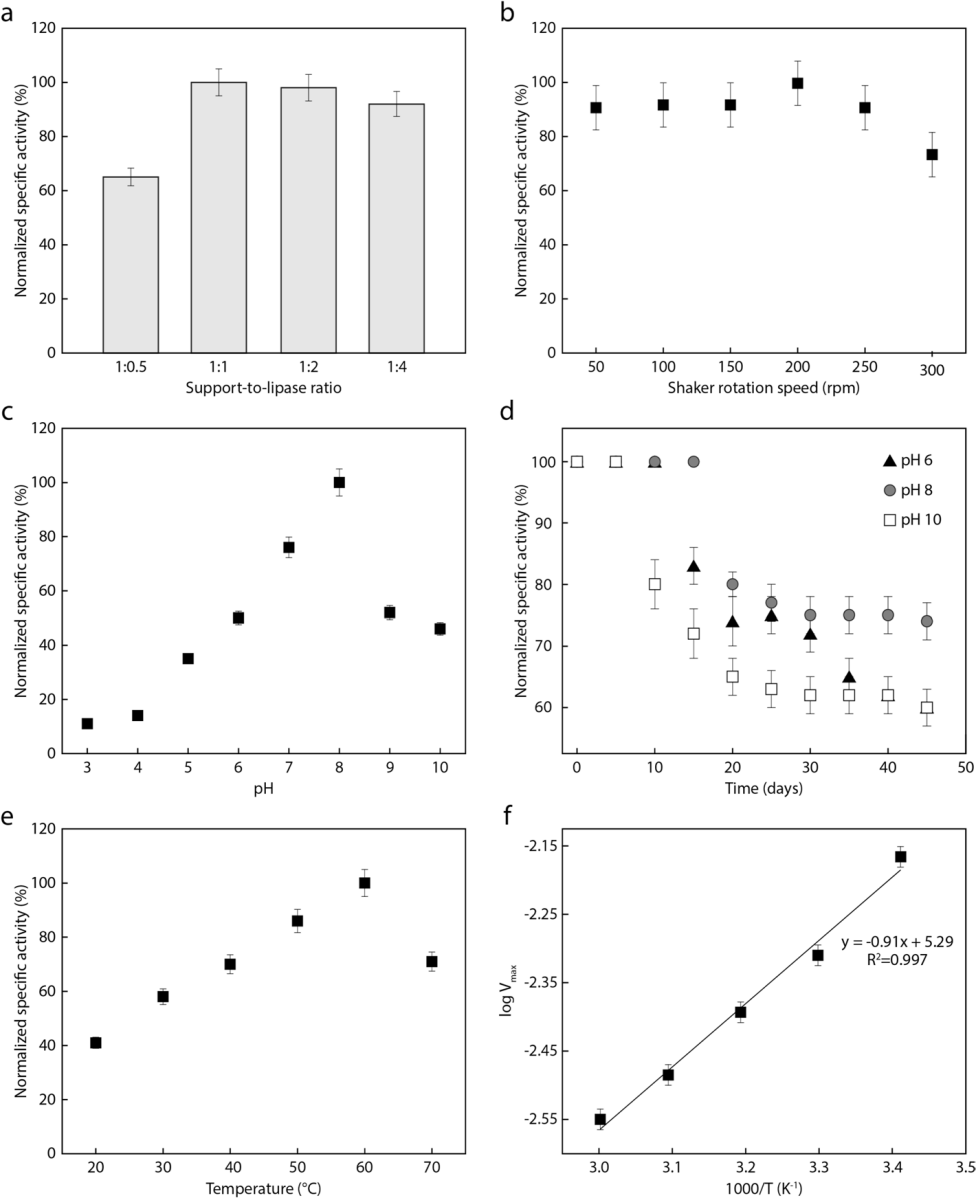
Normalized specific activity as a function of the **a** support-to-lipase (ST–DVB:LBc) ratio, **b** shaker rotation speed, **c** pH, **d** time (for different pH levels), **e** temperature.; and **f** Arrhenius plot based on **e**

To ensure an accurate comparison within each parametric study (see Fig. 2), the normalized specific activity was calculated as $\left(SA/{SA}_{\text{max}}\right)\times 100$, where $SA$ is the specific activity and ${SA}_{\text{max}}$ is the highest specific activity obtained for each study. The protein content was determined using the Bradford method [32] with a fixed protein loading of 30 mg·g^−1^. The activity of both free and immobilized lipase was evaluated using the olive oil hydrolysis method [2, 16]. One unit activity (U) was defined as the amount of lipase that hydrolyzes 1 µmol of olive oil per minute. The immobilization yield was calculated as $\left({A}_{I}/{A}_{0}\right)\times 100$, where ${A}_{I}$ is the hydrolytic activity of the immobilized lipase, and ${A}_{0}$ is the hydrolytic activity of the free lipase (1020 ± 12 U·g^−1^).

### Structural Characterization

Fourier transform infrared (FTIR) spectroscopy was conducted using a Bruker Tensor 27 spectrometer to analyze the free lipase (before contact with supports), pure supports, and biocatalysts (both non-activated and chemically activated). The spectra were collected through 32 scans spanning from 600 to 4000 cm^−1^. A zinc selenide ATR accessory with a 45° penetration angle was employed. A Leica DM 2700 M optical microscope, along with Image Pro-Premier software (V.9.2), was used to capture micrographs of the samples. The microscope was operated in transmission mode and bright field, using a magnification of × 50. The software ImageJ® (Version 1.49) was used to determine the particle size distribution of the ST–DVB supports in their bare state, after treatment with alcohol, and after immobilization. For further microstructural characterization, scanning electron microscopy (SEM) images were taken at × 100 magnification using a Zeiss LEO 440 with an Oxford detector (model 7060). The electron beam was operated at 15 kV with current intensities of 200 pA and 50 pA to ensure precision. All samples underwent gold-sputtering to enhance conductivity. Multiple observations were made under the same conditions to ensure the reproducibility of the resulting microstructures.

### Synthesis of Pear Aroma Through Esterification and Lipase Reuse

The biocatalysts, both non-activated and chemically activated under optimal conditions, were evaluated for their effectiveness in synthesizing pear aroma. The experiments were carried out in 100-mL closed flask reactors, each containing 30 ± 0.5 mL of a substrate mixture consisting of 1-hexanol, acetic acid, and heptane as solvent. For each experiment, 1 g of biocatalyst was used. The effects of different ratios of acetic acid to alcohol (1:4, 1:3, 1:2, 1:1, 2:1, 3:1, and 4:1) on the esterification reaction were investigated at 100 mM. The molar ratio was further varied to 300 mM and 500 mM for optimization. The reactions were performed at a stirring speed of 200 ± 5 rpm, a temperature of 60 ± 2 °C, and atmospheric pressure (101.325 Pa) for up to 24 h. To remove the water produced during esterification, 10 wt.% molecular sieves were used. The progress of the esterification was monitored by sampling the reactor contents and quantifying the consumption of hexyl alcohol and the formation of the esters using gas chromatography (Simple Chrom), as reported by previous work [16]. For the analysis, 1 µL of diluted reaction aliquots was injected into a DB-05 capillary column (J&W Scientific). The injector and detector temperatures were set to 200 °C and 250 °C, respectively. A calibration curve was prepared using standard solutions of hexyl acetate in heptane, with concentrations ranging from 0.05 to 0.25%. The residual acetic acid was measured by titrating aliquots diluted in ethanol with 0.05 M KOH, using phenolphthalein as an indicator [1]. The hexyl acetate yield was calculated as a function of the remaining acetic acid in the reaction.

After the esterification reaction, the enzyme was recovered from the reaction mixture by vacuum filtration using a Buchner funnel. To remove all residual substances from the support, the biocatalysts were thoroughly rinsed with cold hexane and allowed to dry under static conditions for 24 h at room temperature (27 ± 2 °C) before being reused in a new reaction. The biocatalyst was added to fresh substrate to start a new reaction, and this process was repeated for 12 cycles. The operational half-life of the immobilized lipase was estimated based on its residual activity across multiple reaction cycles. A first-order deactivation model was used to fit the activity decay to the expression $A\left(i\right)={{A}_{0}e}^{-{k}_{d}\bullet i},$ where $A\left(i\right)$ is the residual activity of the in the cycle, ${A}_{0}$ is the initial activity, and ${k}_{d}$ is the deactivation constant. The half-life cycle ${i}_{1/2}$ was obtained by setting $A\left({i}_{1/2}\right)=$ 0.5 ${A}_{0}$, which yields ${i}_{1/2}=\frac{\text{ln}(2)}{{k}_{d}}$. The activity data for each condition (non-activated ST–DVB, TX-activated, and PEG/TX-activated ST–DVB) were fitted independently.

### Statistical Analysis

To ensure reproducibility, three independent experiments were conducted for each analysis, and the results are presented as means ± standard deviations. Statistical significance was assessed using Student’s *t*-test, and differences were considered statistically significant at *p* < 0.05.

## Results and Discussion

### Influence of Pretreatment with Ethanol

Optical microscopy was used to examine the particle size distribution of the support during the three key stages of lipase adsorption: prior to contact with lipase, after immersion in a 95% (v/v) ethanol solution, and after lipase immobilization. Image analysis of the optical microscopy images revealed that the bare ST–DVB microspheres have an average diameter of 391 ± 70 µm. Upon exposure to ethanol, the size of the ST–DVB spheres decreased to 349 ± 130 µm, which is in line with previous work [33]. The average diameter increased to 383 ± 89 µm after immobilization.

The ethanol pretreatment step was found to be crucial for the LBc stability. The hydrolytic activity of LBc immobilized without ethanol pretreatment was 137 U·g^−1^, which decreased by ~ 50% after 7 days and dropped to nearly 0 U·g^−1^ after 14 days. This analysis was conducted using the initial immobilization parameters. Because a pronounced reduction prevents the effective synthesis of biocatalysts, the ethanol pretreatment step was adopted for all following experiments.

### Lipase Immobilization Parameters and Morphological Analysis

The impact of the ST–DVB:LBc ratio on the normalized hydrolysis activity is shown in Fig. 2a. The ST–DV:LBc ratio of 1:1 w/w resulted in the highest activity level, with no significant changes upon higher enzyme loading. The addition of more enzymes may not only prevent it from properly reaching the support, but also lead to the immobilization of the lipase in a closed conformation. Optimizing the ST–DVB:LBc ratio is critical for maximizing catalytic potential while avoiding steric hindrance. Previous research [16, 34] has demonstrated that achieving the right enzyme-to-support ratio enhances activity by reducing diffusion barriers to diffusion and maximizing substrate-enzyme interactions on the available surface area.

We also assessed the influence of the orbital shaker rotation speed on the reaction in terms of the hydrolytic activity of lipase. Figure 2b shows no significant differences in the activity of ST–DV–LBc at speeds ranging from 50 to 150 rpm, with the highest activity level observed at 200 rpm. Lower speeds are likely to hinder the enzyme spreading to the support, resulting in reduced lipolytic activity. Conversely, higher speeds can promote lipase denaturation or leaching due to excessive mechanical stresses [35].

Figure 2c reveals the performance of the lipase in the hydrolysis of olive oil as a function of pH. The normalized hydrolytic activity of the free lipase gradually increases with rising pH, peaking at pH 8. Modifying the reaction medium’s pH affects both the enzyme and the support surface, influencing their interaction and, ultimately, the performance of the immobilized lipase. pH also significantly impacts the biocatalyst stability. Variations often lead to conformational changes in enzyme structure, affecting stability and functionality. Depending on the pH, the lipase may experience changes in its structure and activity. Certain pH levels can cause denaturation or deactivation, while others can provide more stability and preserve activity. Both free and immobilized lipase results emphasize that maintaining enzymatic activity is crucial for biotransformation processes. Previous work has shown that milder conditions, especially near neutral pH, exhibit greater effectiveness in sustaining the activity of enzyme [27, 36]. To determine the optimal pH level for the stability of lipase activity, we monitored the hydrolysis activity of immobilized LBc over 45 days at pH 6, 8, and 10. Figure 2d shows that the normalized activity plateaued at 75% for pH 8, and at 60% for pH 6 and pH 10.

The effect of temperature on the normalized lipolytic activity was investigated from 20 to 70 °C. As shown in Fig. 2e, the highest activity for ST–DV–LBc was obtained at 60 °C, with an approximate 25% reduction at 70 °C. The use of ST–DVB in immobilizing lipases from *Candida rugosa* and *Candida antarctica* has shown promising results at temperatures higher than those optimal for free lipase, as previously documented [30, 37]. Lipase reactions are generally studied at temperatures below 70 °C, as lipolytic activity decreases with increasing temperature. Excessive energy disrupts the tertiary structure of the active site, deactivating the enzyme. Thus, the temperature plays a crucial role in the reaction environment, as highlighted by Moreira et al. [1]. Dizge et al. [27] reported optimal temperatures between 40 and 50 °C for *Thermomyces lanuginosus* lipase covalently immobilized on ST–DVB, and 50 °C for the free enzyme. Although the immobilized system retained approximately 35% of its initial activity at 80 °C, demonstrating superior thermal stability compared to the free form, it also exhibited a lower peak activity and a narrower optimal temperature range than that observed in our study.

We analyzed the activation energy of ST–DV–LBc using an Arrhenius plot, a procedure normally conducted to determine the energy rate required to convert a specific substrate into a product [38]. An activation energy of 7.57 ± 0.2 kJ.mol^−1^ was obtained from Fig. 1f, which is lower than the value reported by Moreira et al. [1] for the immobilization of *Burkholderia cepacia* lipase encapsulated in sodium alginate (18.04 kJ.mol^−1^). It is worth noting that even a small increase in temperature can have a significant impact on the reaction rate, as indicated by the low activation energy values. This is because a catalyst reduces the energy required for a reaction. Thus, lower activation energy values correspond to higher reaction rates. These findings emphasize the importance of understanding the lipase interaction with ST–DVB. The analyses in Fig. 2 identified a support-to-lipase ratio of 1:1, a pH of 8, a rotation speed of 200 rpm, and a temperature of 60 °C as optimum conditions. By combining these parameters, a hydrolytic activity of 247 ± 12 U·g^−1^ was obtained.

**Fig. 3:**
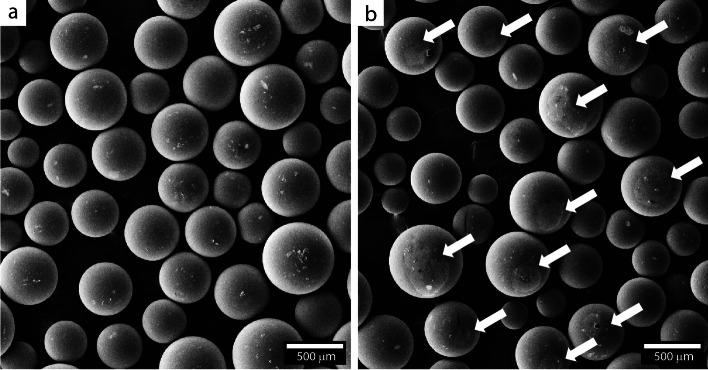
Scanning electron micrographs of ST–DVB particles **a** before and **b** after lipase immobilization conducted under optimized conditions (1:1 support-to-lipase ratio, pH 8, 200 rpm rotation speed, and 60 °C)

To confirm the presence of lipase on the ST–DVB supports, the scanning electron micrographs shown in Fig. 3 were taken before and after immobilization carried out under the optimized conditions identified in Fig. 2. While the white arrows in Fig. 3b highlight regions where the lipase partially covers the support surface, the remaining regions may still contain adsorbed lipase at insufficient levels to produce the contrast required for detection. Additionally, a comparison of the particles in Fig. 3a and b reveals that their shape remained unchanged. This is consistent with Bento et al. [29], who reported similar results following the immobilization of *Candida rugosa* lipase on magnetized ST–DVB supports. The other immobilization conditions examined in Fig. 2 yielded similar micrographs that are not shown here for simplicity.

###  Activation with Chemical Additives

**Fig. 4:**
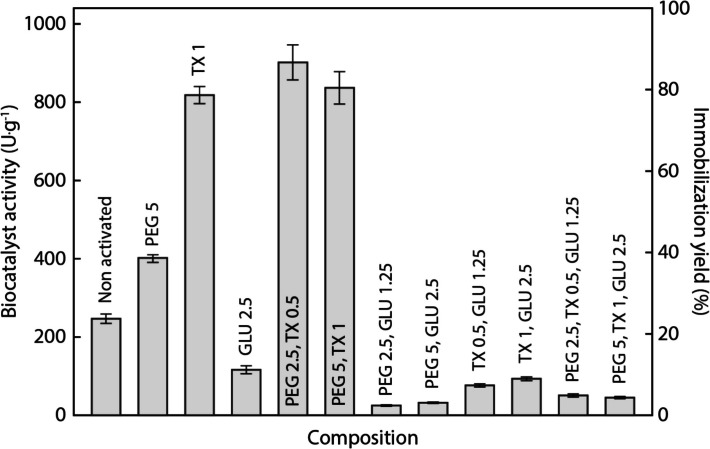
Influence of different chemical additives and their combinations on the biocatalyst activity and immobilization yield taking the activity of the free lipase as reference

The impact of activating ST–DVB supports with polyethylene glycol 1500 (PEG), Triton X-100 (TX), and glutaraldehyde (GLU) on the immobilization of *Burkholderia cepacia* lipase (LBc) was thoroughly investigated with the goal of exploring potential synergies between them. Figure 4 illustrates the biocatalyst activity and immobilization yield resulting from support activation using different additive combinations. The hydrolytic activity of 247 ± 12 U·g^−1^, obtained using the optimal immobilization parameters (Fig. 2), was used as a baseline for assessing the efficacy of each treatment.

Among the tested additives, the ones containing GLU exhibited a pronounced negative effect on enzymatic activity compared to the activity of the non-activated support (ST–DVB–LBc). While GLU is known for enhancing immobilization stability and providing structural protection by promoting the formation of covalent bonds between the enzyme and the support [9, 18, 19, 21–23], our results diverged from those expectations. The observed decrease in catalytic efficiency suggests that excessive cross-linking or structural modification of the enzyme may have occurred, potentially obstructing active sites and limiting enzyme flexibility. The detrimental effects of GLU were evident under all experimental conditions, which can be attributed to reaction conditions such as temperature, pH, time, and the GLU concentration [35, 39].

Pre-treatment of ST–DVB with 5% PEG prior to lipase immobilization increased lipolytic activity (402 ± 10 U·g^−1^) compared to that obtained using non-activated support (247 ± 12 U·g^−1^). According to Perna et al. [25], PEG stabilizes lipase by reducing the solution’s dielectric constant, enhancing dielectric and hydrophobic interactions between the enzyme’s functional groups. PEG also fills structural voids, minimizing the conformational flexibility of lipase and increasing resilience under adverse catalytic conditions [40]. These findings align with those of Vilas Bôas et al. [20], who reported improved esterification efficiency with *Rhizopus oryzae* lipase immobilized using PEG.

A TX concentration of 1% yielded a pronounced increase of enzymatic activity (818 ± 22 U·g^−1^). Combinations of TX with other additives were also investigated, and the highest activity was achieved with a combination of 2.5% PEG and 0.5% TX (901 ± 25 U·g^−1^), highlighting a synergistic effect that enhanced overall catalytic performance. Similar results were reported in Perna et al. [25] and Mesa et al. [26], where the presence of TX improved the catalytic efficiency of the *Candida rugosa* and *Thermomyces lanuginosus* lipases. This improvement can be associated with shifts of the conformational equilibrium of lipases to their open form and the dissociation of bimolecular aggregates, thus facilitating the attachment to hydrophobic supports [25].

### Structural Characterization of Lipase and Chemically Activated Biocatalysts

**Fig. 5:**
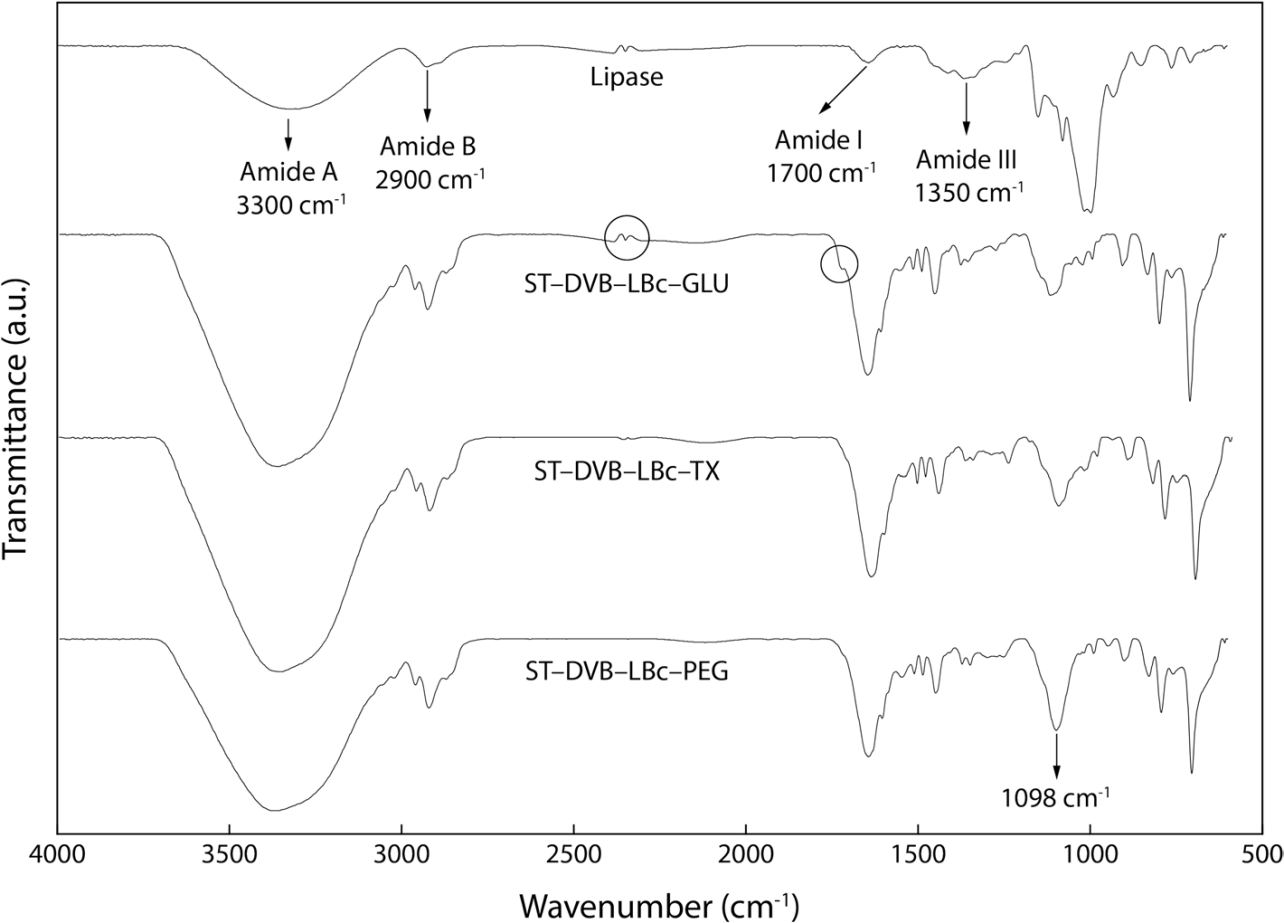
Fourier transform infrared (FTIR) spectra of pure *Burkholderia cepacia* lipase and biocatalysts modified with GLU, TX and PEG. The circles in the spectra highlight specific positions in the ST–DV–LBc–GLU spectrum

Figure 5 compares the free lipase with biocatalysts containing GLU, PEG, and TX individually, highlighting the main modifications in functional groups. The free lipase exhibits a typical protein spectrum, marked by the absorption bands corresponding to amide A (~ 3300 cm^−1^), amide B (~ 2900 cm^−1^), amide I (~ 1700 cm^−1^), and amide III (~ 1350 cm^−1^). These observations align with the findings of Rajan, Benesh, and Nampoothiri [41].

The FTIR spectra of the biocatalyst with GLU show a slight change in the peak at ~ 1700 cm^−1^, associated with the C-N bond characteristic of GLU [41]. Additionally, the lipase peak at ~ 2500 cm^−1^ remains unchanged after immobilization. All biocatalyst spectra display a clear O–H stretching vibration at ~ 3300 cm^−1^. Noticeable distinctions emerge when comparing the spectra of the biocatalysts treated with additives to that of the free lipase, particularly in the amide regions, with differences in intensity and alterations in vibration patterns at 2900 cm^−1^, 1700 cm^−1^, and 1350 cm^−1^. Despite these differences, no significant changes were observed in the functional groups of the analyzed biocatalyst. This suggests that the additives did not induce significant structural or chemical modifications.

The ether group (C–O–C) peak at 1098 cm^−1^ in the ST–DV–LBc–PEG spectrum is more pronounced compared to the free lipase and other biocatalysts. The presence of ether groups suggests a strong connection between the lipase and the support, which could have important implications for the biocatalyst’s stability and efficiency. This supports the results obtained in Fig. 4.

According to Correa et al. [2], lipase immobilization leads to a significant alignment of vibrational peaks between the free lipase and the ST–DVB support. Specifically, the peaks associated with the vibration frequencies near amides showed variations in intensity. It is possible that overlaps have occurred in the same region, as indicated by the more pronounced characteristic bands of the aromatic ring and CH_2_ of pure ST–DVB. Nevertheless, the significant increase in bands for the biocatalysts compared to the pure lipase observed in this study indicates that the physical adsorption immobilization technique is effective with all chemical additives.

### Synthesis of Hexyl Acetate

**Fig. 6:**
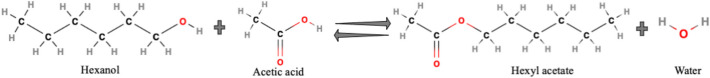
Schematic representation of reaction to synthesis of the pear flavor

A schematic representation of the esterification reaction to produce hexyl acetate from 1-hexanol and acetic acid, using heptane as the solvent, is shown in Fig. 6.

Separate reactor flasks, each containing 1 g of biocatalyst, were prepared with the non-activated biocatalysts, taken as a reference, and the chemically activated biocatalysts yielding optimal conditions (Fig. 4). The biocatalysts used in the experiment exhibited varying yields: 24% for ST–DV–LBc, 80% for ST–DV–LBc–TX, and 88% for ST–DV–LBc–PEG/TX. A study by Karra-Châabouni et al. [42] demonstrated the beneficial impact of a solvent on the equilibrium of hexyl acetate synthesis, which could enhance the transfer of the ester into the organic phase when immobilized *Staphylococcus simulans* lipase is used.

**Fig. 7:**
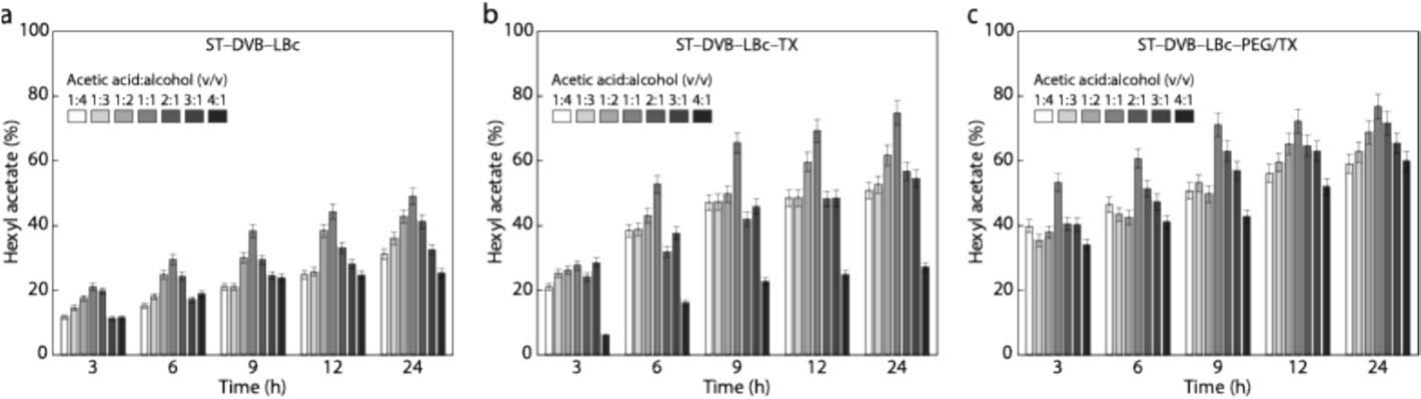
Influence of the ratio of acetic acid to 1-hexanol on the hexyl acetate synthesis using **a** ST–DVB–LBc, **b** ST–DVB–LBc–TX, and **c** ST–DVB–LBC–PEG/TX. The synthesis was conducted at 100 mM, 60 °C, 200 rpm, pH 8, and using 1 g of the biocatalysts

Figure 7 shows the effect of the v/v ratio of acetic acid to 1-hexanol on the hexyl acetate production of immobilized *Burkholderia cepacia* lipase. The ideal synthesis of hexyl acetate is achieved with the equimolar 1:1 ratio for all biocatalysts, while a reduction in ester production is seen upon excess of 1-hexanol. The excess alcohol likely interferes with the lipase’s active site, reducing its efficiency, and limits the availability of acetic acid, impeding the diffusion of reactants to the enzyme’s active site. Similarly, the excess acetic acid inhibits the hexyl acetate synthesis by both the non-activated and chemically activated biocatalysts. The 1:1 ratio for hexyl acetate esterification remains optimal over a 24-h period. Maintaining a careful balance of proportions prevents enzymatic inhibition, enabling efficient interaction between *Burkholderia cepacia* lipase and the substrates upon exposure to 1-hexanol and acetic acid.

Figure 7 also reveals a noticeable improvement in ester conversion when the biocatalyst is chemically treated with TX (Fig. 7b) and PEG/TX (Fig. 7c). These additives played a key role in immobilizing the lipase and facilitating the production of pear aromatic esters due to their strong interaction with 1-hexanol and acetic acid. PEG appears to enhance enzymatic activity and substrate interaction by creating a stable hydrophilic environment, while TX improves substrate solubility and availability through its surfactant properties [10, 20, 26].

**Fig. 8:**
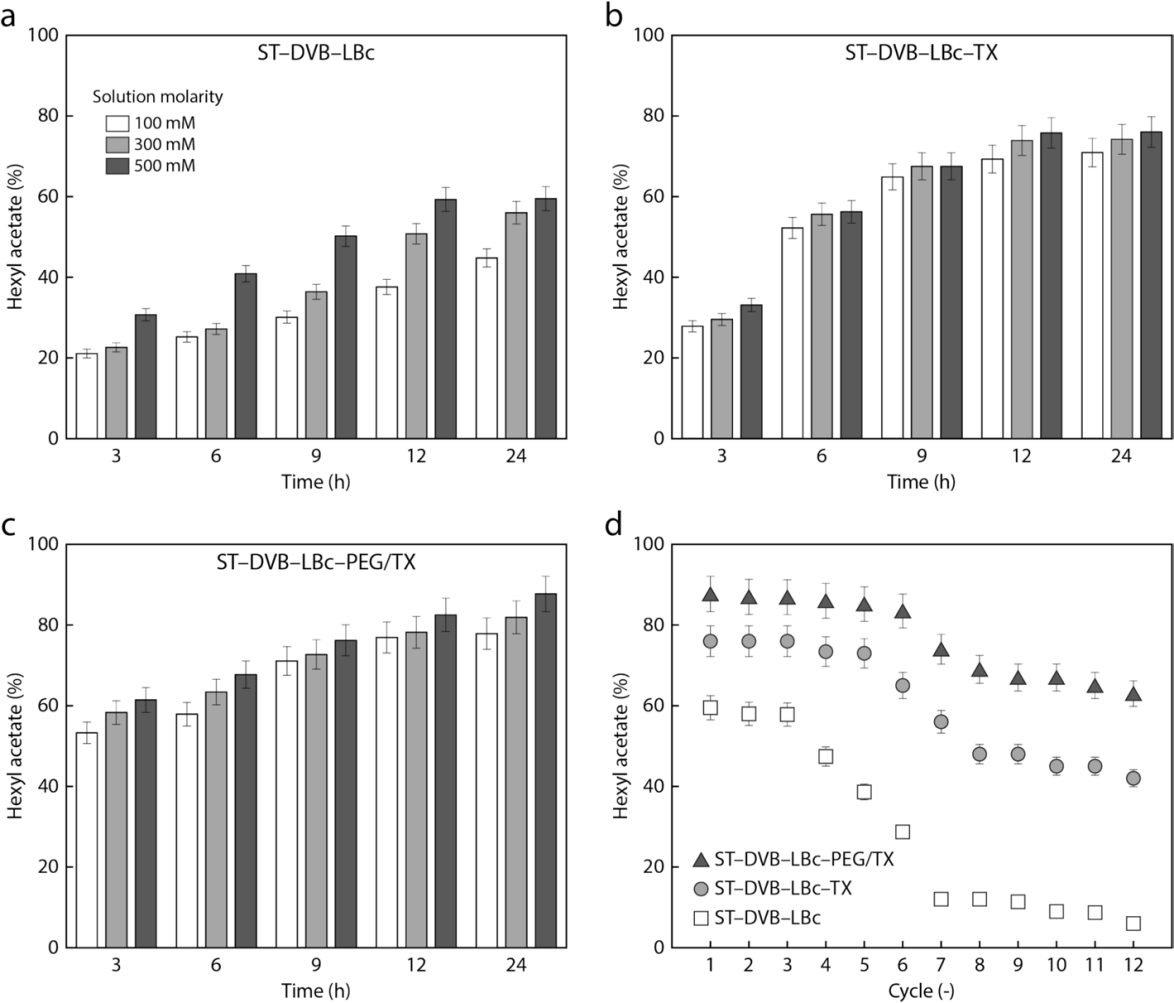
Time evolution of hexyl acetate yield for different molar concentrations and for the biocatalyst **a** ST–DVB–LBc, **b** ST–DVB–LBc–1%TX, and **c** ST–DVB–LBc–2.5%PEG/0.5%TX. The synthesis was conducted with an acetic acid:alcohol ratio of 1:1, at 60 °C, 200 rpm, pH 8, and using 1 g of biocatalyst. **d** Effect of biocatalyst reuse on the hexyl acetate yield

Figure 8 shows a series of experiments conducted to investigate the effects of different concentrations on the esterification process for synthesizing the pear aroma. The biocatalyst exhibited superior performance at a 500 mM concentration with non-activated support, as shown in Fig. 8a. The enhanced availability of substrates increases the frequency of collisions between reactive molecules, accelerating the reaction rate. By carefully adjusting the molarity, optimal conditions for increasing hexyl acetate production were achieved while maintaining high enzyme catalytic efficiency and avoiding product inhibition or lipase denaturation.

Figure 8b and c demonstrate that ST–DVB activated with 1% TX, and with 2.5% PEG and 0.5% TX are effective biocatalysts for pear aroma synthesis compared to the non-active biocatalyst (Fig. 8a). The interaction between PEG and TX during ST–DVB activation likely preserves the lipase’s three-dimensional structure, strengthens its binding to the support, and enhances the support porosity (Fig. 8 c). This creates favorable microenvironments that improve substrate diffusion to active sites and facilitate the removal of bioproducts. In esterification reactions, accumulated products such as esters and water can hinder the activity of lipase.

Figure 8d shows the biocatalyst’s reusability for subsequent pear flavor synthesis. After each reaction cycle, the biocatalysts were collected and evaluated for catalytic activity and stability, emphasizing the sustainability of enzyme processes and the efficient use of resources in industrial applications. Over multiple cycles, the biocatalyst demonstrated consistent activity, highlighting its durability and potential for repeated use without significant performance decline. Enzyme immobilization enhances efficiency and reduces waste in esterification processes, particularly in flavor synthesis, which has both economic and environmental benefits.

After 6 reaction cycles, non-activated ST–DVB and TX- and PEG/TX-activated ST–DVB showed reductions in hexyl acetate yield of 52% (1.47 ± 0.07 mol.L^−1^), 14% (0.46 ± 0.03 mol.L^−1^), and 5% (0.17 ± 0.05 mol.L^−1^), respectively. After 12 cycles, non-activated ST–DVB showed a decrease in flavor yield of 90% (0.29 ± 0.03 mol.L^−1^), while TX- and PEG/TX-activated supports showed slight decreases of 45% (1.62 ± 0.02 mol.L^−1^), and 28% (2.13 ± 0.03 mol.L^−1^), respectively. After 1, 6, and 12 cycles, the hexyl acetate yields of the activated biocatalysts were up to 47%, 191%, and 950% higher than those of the non-activated biocatalyst, respectively. The high yield of pear aroma produced by activated supports indicates that the acetic acid has not inhibited or deactivated the lipase. Previous work reported a 41% hexyl acetate yield with *Staphylococcus simulans* lipase on CaCO_3_ without a solvent, demonstrating that the immobilized lipase could be reused for up to five cycles without any decrease in synthesis activity [42]. Furthermore, the lipase from *Thermomyces lanuginosus* immobilized on the STY–DVB–PGA support exhibited remarkable operational stability, retaining 88% of its initial activity after 10 successive cycles of transesterification using crude canola oil. In contrast, when immobilized on unmodified STY–DVB, celite 545, or silica gel, the enzyme lost its activity entirely within five cycles, likely due to enzyme leaching or structural inactivation caused by solvent exposure and reaction conditions [28]. In terms of operational stability, LBc exhibits half-life values of 3.3, 11.7, and 19.7 cycles for unmodified, TX-activated, and PEG/TX-activated ST–DVB, respectively, confirming that chemical activation significantly extends the functional lifespan of the biocatalyst under reuse conditions. These findings confirm the conclusions in Bento et al. (2017) [29] regarding the benefits of support modification. In their study, *Candida rugosa* lipase immobilization on ST–DVB led to an increased thermal half-life at 60 °C, demonstrating improved resistance to temperature-induced deactivation. Similarly, our results show that ST–DVB activated not only contributes to thermal stabilization but also significantly enhances the operational stability of the biocatalyst, as reflected by its extended reuse over multiple reaction cycles under solvent-free conditions. While Bento et al. [29] focused on thermal deactivation kinetics under constant temperature stress, the present study complements that perspective by highlighting prolonged catalytic performance across repeated cycles, reinforcing the effectiveness of support activation strategies in improving biocatalyst longevity and functionality.

## Conclusion

This study highlights the potential of chemical additives to enhance the stability and performance of immobilized *Burkholderia cepacia* lipase. The enzyme was immobilized on ST–DVB supports through physical adsorption following pre-treatment with ethanol. Optimal immobilization conditions were achieved with a support-to-lipase ratio of 1:1, pH 8, under agitation at 200 rpm and 60 °C. The presence of lipase on the supports was confirmed by the observation of characteristic amide bands A, B, I, and III in the spectral regions around ~ 330 cm^−1^, ~ 2900 cm^−1^, ~ 1700 cm^−1^, and ~ 1350 cm^−1^, respectively.

To further enhance immobilization efficiency and biocatalyst stability, activation treatments using glutaraldehyde (GLU), polyethylene glycol 1500 (PEG), and Triton X-100 (TX) were investigated. The application of TX, as well as combinations of PEG and TX, resulted in biocatalysts with significantly higher activity compared to the non-activated biocatalyst. The best performance was achieved with an activation treatment of 2.5% (v/v) PEG and 0.5% (v/v) TX.

The long-term stability and reusability of the activated supports were assessed by using them in the synthesis of hexyl acetate. The synthesis was monitored over successive operational cycles, and results showed that the hexyl acetate yield of the activated biocatalysts were up to 47%, 191%, and 950% higher than those of the non-activated biocatalyst after 1, 6, and 12 cycles, respectively. This demonstrates the effectiveness of PEG and TX as stabilizing additives in preserving activity and extending the operational lifespan of immobilized *Burkholderia cepacia* lipase. This study opens new pathways for the development of sustainable biocatalytic processes, contributing to a reduction in the environmental impact associated with the industrial production of value-added compounds, such as hexyl acetate, a key component in the pear flavor industry.

## Data Availability

All data for this study are covered in this article.
